# Reducing exposure to high levels of perfluorinated compounds in drinking water improves reproductive outcomes: evidence from an intervention in Minnesota

**DOI:** 10.1186/s12940-020-00591-0

**Published:** 2020-04-22

**Authors:** Gina Waterfield, Martha Rogers, Philippe Grandjean, Maximilian Auffhammer, David Sunding

**Affiliations:** 1 The Nature Conservancy, Arlington, VA 22203 USA; 2 Department of Environmental Health, Harvard T.H. Chan School of Public Health, Boston, MA 02115 USA; 3 Department of Public Health, University of Southern Denmark, Odense, Denmark; 4 Department of Agricultural and Resource Economics, University of California Berkeley, Berkeley, CA 94720 USA; 5 National Bureau of Economic Research, Cambridge, MA 02138 USA

**Keywords:** Birth weight, Fertility, Perfluorocarbons, Pregnancy outcome, Preterm birth, Water pollution

## Abstract

**Background:**

Per- and polyfluoroalkyl substances (PFASs) have been detected in drinking water supplies around the world and are the subject of intense regulatory debate. While they have been associated with several illnesses, their effects on reproductive outcomes remains uncertain.

**Methods:**

We analyzed birth outcomes in the east Minneapolis-St. Paul metropolitan area from 2002 to 2011, where a portion of the population faced elevated exposure to PFASs due to long-term contamination of drinking water supplies from industrial waste disposal. Installation of a water filtration facility in the highly contaminated city of Oakdale, MN at the end of 2006 resulted in a sharp decrease in exposure to PFASs, creating a “natural experiment”. Using a difference-in-differences approach, we compare the changes in birth outcomes before and after water filtration in Oakdale to the changes over the same period in neighboring communities where the treatment of municipal water remained constant.

**Results:**

Average birth weight and average gestational age were statistically significantly lower in the highly exposed population than in the control area prior to filtration of municipal water supply. The highly exposed population faced increased odds of low birth weight (adjusted odds ratio 1.36, 95% CI 1.25–1.48) and pre-term birth (adjusted odds ratio 1.14, 95% CI 1.09–1.19) relative to the control before filtration, and these differences moderated after filtration. The general fertility rate was also significantly lower in the exposed population (incidence rate ratio 0.73, 95% CI 0.69–0.77) prior to filtration and appeared to be rebounding post-2006.

**Conclusions:**

Our findings provide evidence of a causal relationship between filtration of drinking water containing high levels of exposure to PFASs and improved reproductive outcomes.

## Background

Per- and polyfluoroalkyl substances (PFASs) are a class of industrial chemicals that have been manufactured in the U.S. since the 1950s. PFASs have been used in a variety of consumer and industrial products, including water repellant fabric treatments, non-stick cookware, and fire-fighting foams, for their surfactant properties and resistance to degradation [1]. These qualities also mean that PFASs are persistent in the environment and accumulate in the human body. As a result, they have been found in human blood samples from populations all over the world and are increasingly being detected in municipal drinking water supplies [2, 3]. In the face of public concern over mounting evidence that PFASs are associated with adverse human health outcomes, the U.S. Environmental Protection Agency recently released a plan to revisit federal exposure standards [4].

Perfluorooctanoic acid (PFOA) and perfluorooctane sulfonic acid (PFOS) are the most widely studied compounds within the class of PFASs and have been associated with a range of adverse health effects, including cancer [1]. Some studies have identified relationships between PFAS exposure and reproductive outcomes, although the evidence regarding the pathway and significance of associations is mixed. The most consistent finding is a negative association between maternal serum-PFOA or PFOS concentrations and infant birth weight [5–9], although not all studies have found evidence of this relationship [10, 11]. A meta-analysis of nine epidemiological studies showed a highly significant, though fairly small, decrease in birth weight at elevated PFOA exposure [12], and this outcome is considered as an established effect by the Agency for Toxic Substances and Disease Registry (ATSDR) [1]. Prior studies also provide some evidence of an association between maternal serum-PFAS concentrations and gestational age at birth [5, 7, 13, 14].

ATSDR also concludes that elevated exposures to PFOS and PFOA are associated with decreased fertility, as some human epidemiological studies have reported a negative association between serum-PFAS concentrations and fecundity, measured through time-to-pregnancy (TTP) [15, 16], although results may be affected by parity [17–19]. Most of this research has focused on maternal serum concentrations, although paternal exposure may also be relevant [20, 21].

This paper provides new evidence of the effect of PFOA and PFOS exposure on reproductive outcomes. Our study focused on residents of Oakdale, Minnesota in the east Minneapolis-St. Paul metropolitan area, where elevated concentrations of PFOA and PFOS were detected in municipal water supplies as a result of industrial disposal in nearby landfills beginning in the 1950’s [22] (see Additional file 1 for a map indicating the location of contamination source sites and Additional file 2 for maps of recent levels of groundwater contamination in the area). Drinking water supplies in certain other nearby communities, in particular Cottage Grove, Lake Elmo, and Woodbury, are also known to have been contaminated by PFAS, but exposure was less consistent across the population due to greater reliance on private domestic wells. In November 2006, following discovery of the contamination, a granular activated charcoal (GAC) water filtration facility was constructed that reduced PFAS concentrations in Oakdale’s municipal water below applicable guidelines [22]. More limited interventions occurred in other communities, with some private domestic well owners provided with bottled water or connected to municipal systems.

The intervention in Oakdale constitutes a classic “natural experiment”, a common setting used to evaluate causal relationships when randomized control trials are infeasible [23, 24]. In natural experiments, individuals are assigned to treatment and control groups by some process that is external to the individuals themselves and to the researcher. Ideally, the treatment condition also changes exogenously over time in a non-uniform manner across subsets of the population. Changes in the outcome of interest can then be compared between these subsets, implicitly controlling for a broad array of confounding factors that cannot be controlled for in a cross-sectional analysis. Confounding may still be a concern, but only if the omitted variables change over time in a manner that differs across treatment and control groups.

In Oakdale, exposure to PFAS declined dramatically in 2006 due to the installation of the filtration system, while exposure in neighboring unaffected communities was largely unchanged. We compared outcomes among residents of Oakdale to those in the control communities before and after the change in PFAS exposure using a differences-in-differences approach. This approach implicitly controls for any unobserved differences between Oakdale and the control communities, mitigating concerns regarding omitted variable bias. With the exception of one study that modeled serum levels due to PFOA exposure through drinking water contamination in the Ohio Valley region [14], prior studies have estimated only associations between serum PFAS concentrations and birth outcomes, which may be impacted by confounding factors [25]. For example, individuals with poor kidney function may be less able to remove PFAS from the bloodstream, and their poor kidney function may also affect birth outcomes [26].

Our aim in this study was to adapt a method relied on heavily in social sciences and public policy evaluation [24] to provide further evidence of the causal relationship between PFAS exposure and reproductive outcomes, namely birth weight, gestational age, the risks of low birth weight and preterm birth, and the general fertility rate (GFR).

## Materials and methods

For our analysis, we used the population of individual-level birth record data spanning 2002 to 2011 (5 years pre- and post- installation of the filtration facility in Oakdale) for the entirety of Washington County and portions of neighboring counties. A summary of maternal and newborn characteristics, as well as zip code level demographics, for our study population is provided in Table 1.

**Table 1:**
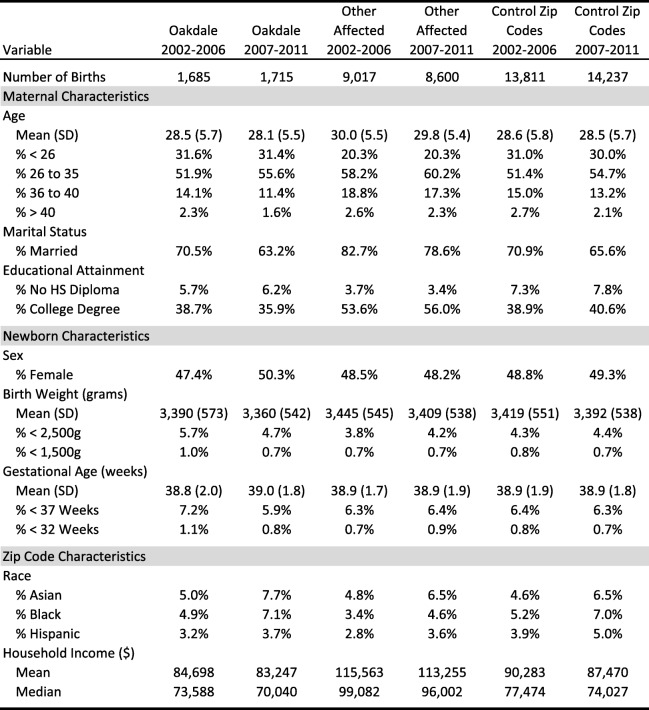
Summary of maternal newborn, and zip code characteristics by community

### Study population and data

We relied on individual-level birth record data for over 48,000 births collected by the Minnesota Department of Health (MDH), representing all singleton live births between 2002 and 2011 in all zip codes contained at least partially within Washington county – the county in which Oakdale resides – excluding certain communities for which information regarding PFAS concentrations was insufficient. The birth data included mother’s age, marital status, and educational attainment at time of birth; birth month and year, gestational age, birth weight, and infant sex; and zip codes of maternal residence address.

To account for socioeconomic factors not represented in the birth data itself, we introduced demographic variables from the U.S. Census Bureau’s decennial census and the American Community Survey (ACS). Average household income and racial composition were included at the zip-code level and served as proxies for individual-level maternal characteristics. Values for the years 2002–2009 were linearly interpolated from the 2000 and 2010 censuses, while 2011 values were taken directly from the ACS. While individual-level data would enable more accurate control of demographic changes in the study area over time, these data are unavailable due to privacy concerns.

### Exposure

Exposure to PFAS is approximated by the residential zip code of a mother at the time of birth combined with birth year. Approximately 99% of Oakdale’s population of 27,000 residents were served by the city’s municipal water system. According to 2005 testing, among Oakdale’s nine municipal wells, PFOA concentrations ranged from 0.07 to 0.70 μg/L and PFOS concentrations ranged from a non-detectable concentration to 1.04 μg/L [22]. For reference, the EPA’s current drinking water health advisory levels for PFOA and for PFOS are both 0.07μg/L individually and when concentrations are combined [27, 28]. Certain states, including the State of Minnesota, recommend more stringent standards (MDH’s current health-based values are 0.035 μg/L for PFOA and 0.015 μg/L for PFOS) [29].

Drinking water in certain other nearby communities, including Cottage Grove, Lake Elmo, and Woodbury, are also known to have been contaminated by PFAS but did not install municipal GAC filtration during our study period. Exposure was also less consistent in these other affected communities due to greater reliance on private domestic wells. These communities were therefore considered separately in our analyses. The communities used as our control area have not been found to have detectable levels of PFAS (see Additional file 2) or were beyond the area the Minnesota Pollution Control Agency (MPCA) found necessary for testing due to their distance from known PFAS disposal sites. MPCA also conducts ambient monitoring of PFAS in groundwater across the state [30]. The city of Oakdale is covered fully by a single zip code (55128). The other affected communities of Cottage Grove, Lake Elmo, and Woodbury are covered by a total of seven zip codes. The remaining 22 zip codes that overlap with Washington County constitute the control areas.

Our proxy for PFAS exposure does not reflect differences in individual water ingestion and intakes from private wells or water filtration devices, or consumption of bottled water. Further, this exposure parameter does not reflect the gradual nature of the decline in serum-PFAS concentrations once exposure ceases. Still, residents in the affected areas were known to have elevated serum-PFAS concentrations during the sample period, with geometric mean PFOA and PFOS concentrations of 15.4 ng/mL (range, 1.6–177 ng/mL) and 35.9 ng/mL (range, 3.2–448 ng/mL) respectively, according to volunteer samples obtained in 2008, 2 years after GAC was installed [31]. These means exceed national averages in 2007–2008 by almost 4-fold for PFOA and 3-fold for PFOS [32]. Moreover, the MDH biomonitoring samples were collected approximately 2 years after the treatment facility in Oakdale was brought online, so serum concentrations among this population would have been higher prior to 2006.

### Outcomes

We first evaluated birth weight and gestational age as continuous outcomes, and then studied binary adverse outcomes of birth weight < 2500 g and < 1500 g, and gestational age at birth < 37 weeks and < 32 weeks. The analysis of binary adverse outcomes was made feasible from a statistical standpoint by our relatively large sample size. Table 1 reports the percent of births in each outcome category, by community and period. We also evaluated the GFR by zip code, defined as the number of births per 1000 women aged 15–44 years, with the denominator based on U.S. Census data. To test whether our results were driven by changes in the distribution of age within the population of women aged 15–44, we evaluated the fertility rate specific to four age groups (15–19, 20–24, 25–34, and 35–44).

### Statistical approach

Taking advantage of the Oakdale-wide changes in PFAS exposure, we used a difference-in-differences approach to evaluate all outcomes [33], both continuous and binary. In each model, indicator variables were included for Oakdale in the 5 years before the construction of the GAC facility (2002–2006), Oakdale in the 5 years following the intervention (2007–2011), the other affected communities overall, and for each year to control for common changes over time, as well as for birth month to control for seasonal patterns. The estimated coefficients on the geographic indicator variables reflect differences relative to the control area in the corresponding time period. In all the estimations, the omitted group is the control communities – meaning that no indicator variable was included for zip codes in the control areas.

We estimated all models both with and without individual-level characteristics (infant sex being female, mother’s age, educational attainment, and marital status) and with and without zip-code level characteristics (mean household income and percentages of the population being Asian, Black, or Hispanic). Indicators for maternal drug use and medical risk factors were included as robustness checks, but since these data appear inconsistent over time, results from these estimations are provided in the additional files. Our primary estimating equations are all similarly defined as:

$$ Birth\ Outcome=f\left(\alpha +{\beta}_1{Oakdale}_{2002-2006}+{\beta}_2{Oakdale}_{2007-2011}+{\beta}_3 Other\ Affected+{\beta}_4 Maternal\ Charateristics+{\beta}_4 Zip\  Code\ Characteristics+\gamma Birth\ Years+\theta Birth\ Months+\epsilon \right) $$

The coefficients of interest are *β*_1_ and *β*_2_, which indicate the difference in mean or probability of an outcome in Oakdale relative to the control communities before and after filtration respectively. The difference between these two coefficients is therefore indicative of the effect of filtration. The vectors of individual maternal characteristics and zip code-level characteristics control for time-variant differences across communities. The vectors of birth month and birth year effects control for changes over time and season that are common across communities.

Continuous birth outcomes were modeled using ordinary least squares regression with standard errors clustered at the zip-code level. Binary individual-level outcomes (low and very low birth weight, pre-term and early pre-term birth) were modeled using logistic regression estimated via maximum likelihood, with standard errors clustered at the zip-code level. For binary individual-level outcomes, results are reported as odds ratios, which reflect the increased or decreased odds of the specified birth outcome for mothers living in Oakdale relative to the control areas.

General and age-specific fertility rates were estimated by Poisson regression via maximum likelihood using robust standard errors [34]. The number of women within the relevant age range was included as a covariate with the coefficient constrained to one. Since fertility rates are defined for some population, we used zip codes as our unit of analysis. All covariates are therefore aggregated to the zip-code level. For general and age-specific fertility rates, results are reported as incidence rate ratios, which reflect the increased or decreased rates of fertility in Oakdale relative to the control areas during the same time period.

## Results

**Fig. 1:**
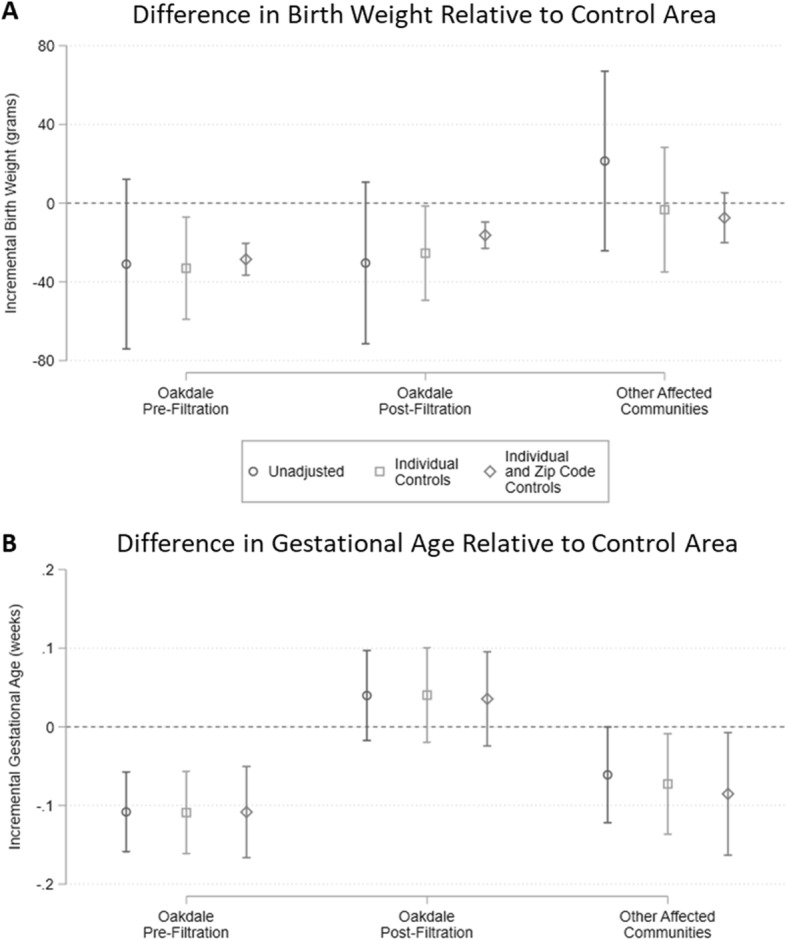
Mean birth weight and gestational age in highly exposed communities relative to control. Bars depict coefficients on community indicator variables from ordinary least squares regressions of birth weight in grams (**a**) and gestational age in weeks (**b**). Error bars indicate 95% confidence intervals using standard errors clustered at the zip-code level. Unadjusted models include birth month and year fixed effects only. Models with individual controls also include indicators for female infant, maternal marital status, maternal age (<20 years, 20-24 years, 35-39 years, >39 years), and maternal educational attainment (no high school diploma, college degree or higher). Models with zip-code controls also include mean household income and racial composition (% Asian, % Black, % Hispanic)

The coefficients on the community indicator variables for our continuous birth outcomes are illustrated in Fig. 1. Full estimation results are included in the additional files (see Additional file 3). Based on the model including individual and zip code level controls, which offers the most precise estimates, birth weights were on average 29 g lower in Oakdale than in neighboring unaffected communities in the 5 years prior to the intervention but only 16 g lower in the 5 years following the intervention. Although small relative to mean birthweight and overall variation in the data, both differences were significant at the *p* < 0.01 level, and significantly different from one another. Average birth weights in the other affected communities were 7.0 g lower than those in the control area but with a *p*-value of 0.23. The inclusion of individual and zip-code level characteristics reduced the estimated standard errors and the magnitude of the Oakdale post-filtration coefficient. Inclusion of these covariates also changed the sign of the coefficient estimated for the other affected communities, likely due to differences in socioeconomic characteristics across communities.

Estimates for gestational age demonstrated a similar pattern. Infants born in Oakdale before filtration were born an average of 0.1 weeks earlier than those in the control area, but the difference dissipated in the post-filtration period. Gestational age at birth was 0.09 weeks lower in the other affected communities than in the control area over the entire study period. Again, while these differences were small in magnitude, they were statistically significant at *p* < 0.01 and *p* < 0.05 levels, respectively. As indicated by comparison across the three models, estimated coefficients were insensitive to the inclusion of covariates but more precisely estimated with these additional controls.

In both the birth weight and gestational age regressions, the coefficients on the individual and zip-code level characteristics had the anticipated signs (see additional files). These materials also include results from two additional regressions where maternal drug use and medical risk factors are included in the individual controls. These results are omitted from Fig. 1 because the reporting of these data was inconsistent over time. Nonetheless, when included, drug use and presence of medical risk factors were strongly negatively correlated with the outcome variables. The inclusion of these two additional controls did not, however, affect the estimated coefficients on the pre- and post-filtration Oakdale indicator variables or the indicator for the other affected communities. The limited impact of including these additional controls on the coefficients of interest is likely due to the strong correlation between maternal drug use and medical risk factors with the other included socioeconomic variables, such as education, age, and marital status.

**Fig. 2:**
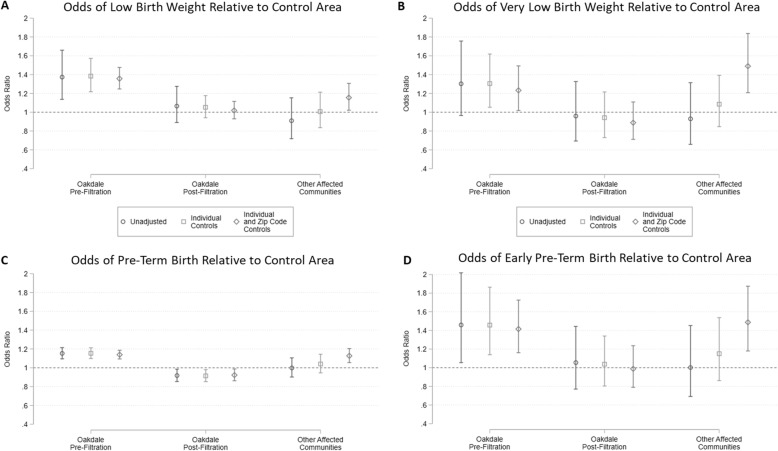
Odds of low birth weight and pre-term birth in highly exposed communities relative to control. Bars depict coefficients on community indicator variables from logistic regressions of binary outcomes: birth weight <2,500g (**a**), birth weight <1,500g (**b**), gestational age <37 weeks (**c**), and gestational age <32 weeks (**d**). Error bars indicate 95% confidence intervals using standard errors clustered at the zip-code level. Unadjusted models include birth month and year fixed effects only. Models with individual controls also include indicators for female infant, maternal marital status, maternal age (<20 years, 20-24 years, 35-39 years, >39 years), and maternal educational attainment (no high school diploma, college degree or higher). Models with zip code controls also include mean household income and racial composition (% Asian, % Black, % Hispanic)

We further estimated the effects of PFAS exposure on the probability of binary birth outcomes known to be associated with adverse long-term developmental consequences. Figure 2 presents the estimated odds ratio (OR) for low birth weight (< 2500 g, panel A) and very low birth weight (< 1500 g, panel B) births in the exposed communities relative to the control area, estimated via logistic regression with and without additional control variables. An OR greater than one for a community signifies that mothers in that community faced increased odds of giving birth to infants of low or very low birth weight relative to the control communities. The estimated ORs from the models that include individual and zip code level characteristics for low birth weight and for very low birth weight in Oakdale pre-filtration were 1.36 and 1.23, respectively. Both were statistically significantly different from one and insensitive to the inclusion of covariates. The Oakdale post-filtration OR was not significantly different from one for either binary birth weight outcome. Estimated ORs for the other affected communities were significantly elevated when both individual and zip code level characteristics were controlled for.

The same models were also estimated for the probability of pre-term birth (< 37 weeks, panel C) and early pre-term birth (< 32 weeks, panel D). Similar patterns hold for pre-term births (Oakdale pre-filtration OR 1.14) and early pre-term births (Oakdale pre-filtration OR 1.42), as were observed for low and very low birth weight. As with the birth weight and gestational age regressions, coefficients on individual and zip-code level characteristics, when included, had the anticipated signs (see Additional files 4 and 5).

To evaluate the effect of PFAS exposure on fertility, we estimated models of the GFR and fertility rates among women in specific age ranges as functions of the same zip-code level characteristics used above. The GFR is defined as the number of births in a given population divided by the number of women in the population of child-bearing age, typically assumed to be women ages 15–44. Age-range specific fertility rates are defined as the number of births to women in that age range, divided by the total number of women in that age range within the population. Evaluation of fertility rates within specific age ranges provides insight into the extent to which observed difference in the GFR are attributable to changes in the age distribution of women within the population.

**Fig. 3:**
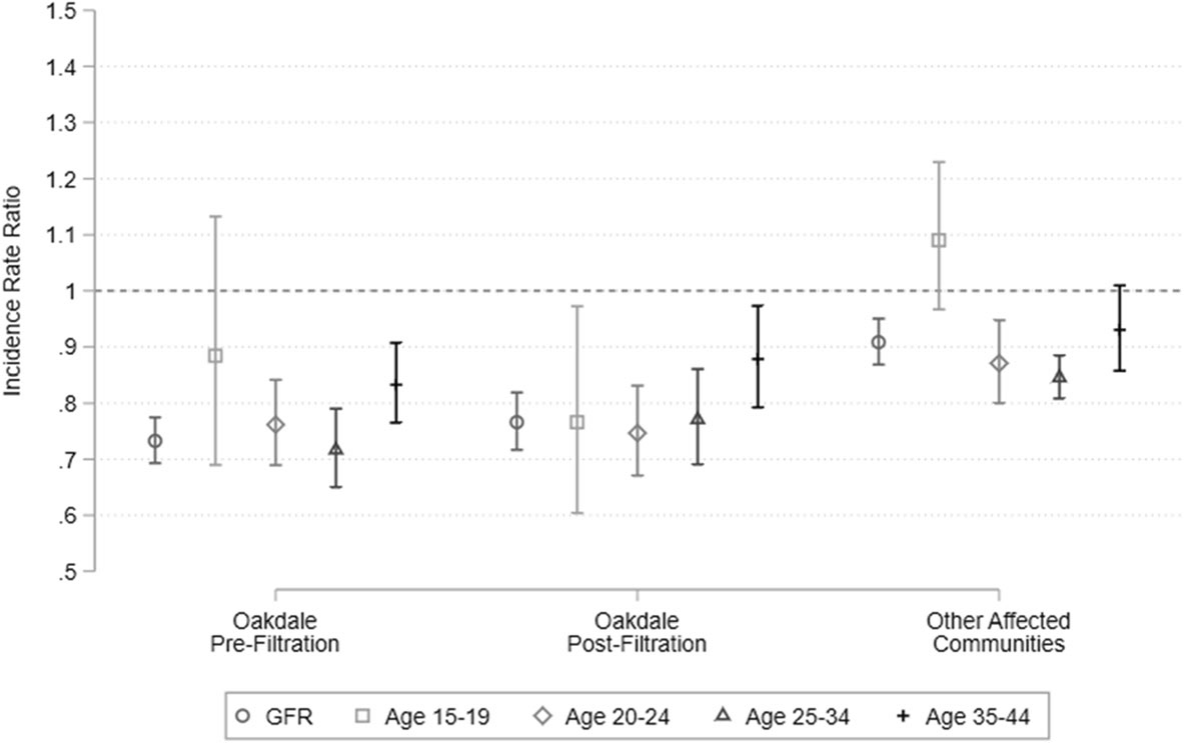
incidence rate ratios for general and age group fertility rates in highly exposed communities relative to control. Bars depict coefficients on community indicator variables from Poisson regressions of the number of births within a zip code, controlling for the number of women between ages 15 and 44, or within each specified age range, with coefficient constrained to 1. Error bars indicate 95% confidence intervals using robust standard errors. Coefficients reported as incidence rate ratios. All models include year fixed effects, mean household income, educational attainment (% HS diploma and % college degree) and racial composition (% Asian, % Black, % Hispanic)

Figure 3 presents the estimated coefficients on the community indicator variables as incidence rate ratios (IRRs), based on models that adjust for zip-code characteristics. Full regression estimates for all models with and without zip-code controls are included in the additional files (Additional file 6). As with the OR, an IRR less than one for a given community indicates that women residing in zip codes in that community had fewer births than did women residing in the control zip codes, after controlling for the number of women in the relevant age range within each zip code. The GFR in Oakdale was equal to 0.73 times the mean GFR in the control zip codes pre-filtration, and 0.77 times the mean GFR in the control zip codes post-filtration. Both estimates are significantly different from one at the *p* < 0.01 level. In the other affected communities, the GFR was 0.91 times that in the control zip codes and significantly different at the *p* < 0.01 level.

The differences in the GFR in the highly exposed communities appears to be driven by births to mothers in the 25–34 age range. Mothers in this age range account for the majority of births in our study population. Women age 25–34 residing in Oakdale gave birth 0.72 times as frequently as did women age 25–34 in the control zip codes pre-filtration, and 0.77 times as frequently post-filtration. Women age 25–34 in the other affected communities gave birth 0.85 times as frequently as did women age 25–34 in the control zip codes between the entire 2002 and 2011 study period.

## Discussion

Our results indicate a modest but statistically significant lower mean birth weight among infants born to mothers that resided in areas with high concentrations of PFOS and PFOA in drinking water supplies. These findings are consistent with the majority of the prior literature that has evaluated these relationships [1, 12, 35]. We also found evidence that PFOS and PFOA exposure is linked to lower mean gestational age, a relationship that has found less consistent support in previous studies [5, 13, 14].

Associations between maternal PFAS exposure and birth weight and/or gestational age are of more concern if they entail a greater likelihood of clinically significant low birth weight (< 2500 g) or preterm birth (< 37 weeks), outcomes which have been associated with adverse long-term developmental consequences for the child [36, 37]. However, few studies have evaluated associations between PFAS and the likelihood of these adverse birth outcomes directly (exceptions include [10, 14, 38]), largely due to sample size constraints given the relative infrequency with which such outcomes occur.

Our analyses suggest that high levels of PFOA and PFOS exposure may lead to substantially increased odds of low birth weight, very low birth weight, pre-term birth, early pre-term birth, and a lower GFR. All of these outcomes are of substantial public health relevance [36–38]. We note, however, that these effects are not necessarily additive. In particular, pre-term birth may lead to an increased risk of low birth weight, such that these two outcomes are correlated. Part of the effect of PFAS exposure on the odds of low birth weight is thus already captured in the effect of PFAS exposure on pre-term birth. PFAS exposure may still be a causal factor in both outcomes though, and our estimates represent the overall effect of exposure on each outcome regardless of the mechanisms through which it acts.. In Oakdale, the area of highest and most consistent exposure before the installation of GAC water filtration, infants were 36% more likely to be born at a weight less than 2500 g and almost 45% more likely to be born prior to 32 weeks, relative to control communities unaffected by the contamination. These differences in outcomes between the highly exposed community and the control area moderated after municipal water filtration began and PFAS exposures were dramatically reduced.

Methodologically, the present study differs from the existing literature in several ways [39]. First, the contamination of drinking water supplies in our study area affected a large population, with approximately 19,000 (roughly 40%) of the birth records in our study being to mothers residing in affected areas. This large study population affords greater statistical power, a feature of particular importance in evaluating the probability of relatively rare birth outcomes such as early pre-term birth.

Second, with the exception of one C8 Science Panel study [14], prior studies have estimated only associations between individual serum-PFAS concentrations and birth outcomes. Such studies may be subject to concerns regarding the influence of unobserved confounding biological factors [25, 26]. For example, individuals with poor kidney function may be subject to slower removal of PFAS from the blood, leading to higher serum concentrations, and may also experience worse reproductive outcomes as a result of poor kidney function. Our proxy for exposure, however, does not rely on measured serum concentrations. By using a difference-in-differences approach, our analysis avoids this concern.

Third, the majority of prior studies are based on samples from general populations with more modest background levels of exposure to PFAS, limiting the range over which any relationships may be identified. Other studies that have estimated associations with high levels of PFAS exposure have been based on populations exposed primarily to PFOA [14, 40]. Residents of Oakdale and the other affected communities were highly exposed to both PFOS and PFOA. Animal-based toxicological studies indicate that PFOS is a significant determinant of adverse birth outcomes including decreased pup weight and survival [1]. The effects of exposure to PFOA and PFOS may also be additive, with certain health-based values specifying joint exposure limits [27].

Fourth, our use of all singleton live births in our study communities over the relevant time period enables a novel evaluation of the effects of PFAS exposure on fertility through analysis of the GFR and age-specific fertility rates. Unlike prior studies of TTP (time-to-pregnancy) that follow a self-selected set of individuals that succeed in conceiving, the research design in the present study does not implicitly omit the impact of PFAS exposure on infertility. Our results provide the first epidemiological evidence that elevated PFAS exposure affects fertility; the GFR in Oakdale was 15 to 25% lower among women in fertile age groups than rates observed in the control communities. While the post-filtration recovery in Oakdale appears to be slower or more modest for the general fertility rate than for the individual adverse birth outcomes evaluated, the estimated coefficients are still higher for the 2007–2011 period than for the 2002–2006 period. The rebound appears to be driven by 25–34-year-old women who represent the largest age category for mothers in our study.

While our study of the GFR cannot disentangle the particular mechanisms through which PFOA and/or PFOS exposure may affect the birth rate, it reflects the outcome of public health interest. Our study is, however, limited by lack of information on contraceptive use and other important choice-based determinants of fertility. To the extent that these variables are not captured by the demographic information included in the analysis and changed differently over time in Oakdale relative to the control communities, our estimates may be subject to confounding.

Fifth, the installation of the GAC filtration facility in Oakdale in 2006 effectively eliminated Oakdale residents’ exposure to non-background concentrations of PFAS. This population-wide change in exposure allows a difference–in-differences comparison that provides a stronger basis for causal inference than a standard cross-sectional cohort-based study, as all confounding factors that do not change over time are implicitly controlled for. A type I error will arise only if omitted factors change at the same time as the intervention. One conceivable confounding factor could be the presence of other contaminants in Oakdale’s municipal water supply with concentrations that were also reduced by GAC filtration. However, extensive monitoring and remediation to address the presence of known contaminants in groundwater in Washington County was undertaken in the 1980s [22].

Given the limitations on demographic information included in our data due to privacy concerns, it could also be the case that the composition of Oakdale’s population changed relative to the surrounding areas pre- and post-installation of the filtration system. Table 1, however, shows that the demographics of Oakdale changed in a similar way to the control zip codes suggesting that the results were not driven by temporal changes in race/ethnicity or income levels. Moreover, inclusion of these zip-code level variables in our models did not change our findings. Similarly, it is unlikely that significant differences in birth order emerged pre- and post-filtration in Oakdale relative to the control areas, especially since our analyses were adjusted for age.

Lastly, the present study is distinct due to the long study period. Exposure outcomes must be evaluated over a relatively long timeframe due to the fact that the half-lives of PFOA and PFOS in human blood have been estimated to be in the range of 2 to 4 years [41]. Maternal serum-PFAS concentrations in Oakdale would therefore remain elevated even after consumption of contaminated water ceased. Our binary classification of this gradual decline, using 2006 as a cutoff point, will tend to attenuate the magnitude and statistical significance of any relationships identified by introducing measurement error in the explanatory variable of interest [42]. Our data do, however, include mothers who moved into Oakdale and the other affected communities after 2006, and average serum concentrations across all residents of Oakdale may have declined at a faster rate than would be suggested by the elimination half-life only. In addition, transplacental passage of PFASs could be faster for recently absorbed PFASs than for accumulated PFAS stores that are bound to albumin or other ligands.

There are several important limitations of the present study. First, our data do not allow for the estimation of a dose-response relationship. Precise individual-level maternal exposure to PFAS in our study population is unknown, and our proxy for exposure is binary, although supported by analyses of serum samples thought to be representative for the community [31, 43]. Second, we were unable to disentangle the effects of PFOA and PFOS from one another, and from the potential effects of other PFASs. In particular, perfluorobutyrate (PFBA) contamination was also widespread in the Washington County area, although the prior literature does not suggest that PFBA is associated with the adverse reproductive outcomes observed. Third, drinking water is not the only exposure pathway for PFAS. Consumption of fish caught from contaminated water bodies is another local source of exposure, and fish consumption advisories due to the presence of PFOS in fish tissues were released only beginning in 2008 [44]. Lastly, our research design and potential annual variation in unobserved factors do not allow for a year-by-year analysis that explicitly accounts for the long half-lives of PFAS in the body. Further follow-up and extended access to covariates will be needed to establish the continued normalization of reproductive parameters in Oakdale.

## Conclusions

PFAS exposure from contaminated drinking water in Oakdale, MN during 2001–2006 was associated with increased odds of low birth weight, pre-term birth and lowered general fertility relative to uncontaminated control populations. Moreover, these differences moderated after installation of a water filtration facility at the end of 2006. This study provides new evidence of a causal relationship between an intervention to reduce elevated levels of exposure to PFASs through drinking water and improved reproductive outcomes.

## Supplementary information

**Additional file 1: Figure A1.** Map of Study Area and PFAS Source Locations.

**Additional file 2: Figure A2.** Current Extent of PFOS and PFOA Contamination of Groundwater in East Metro Area.

**Additional file 3: Table A1.** All Regression Coefficients for Continuous Birth Outcomes.

**Additional file 4: Table A2.** All Regression Coefficients for Low Birth Weight (< 2500 g) and Very Low Birth Weight (< 1500 g) Models, Reported as Odds Ratios.

**Additional file 5: Table A3.** All Regression Coefficients for Pre-term Births (< 37 weeks) and Early Pre-term Births (< 32 weeks) Models, Reported as Odds Ratios.

**Additional file 6: Table A4.** All Regression Coefficients for General and Age Group-Specific Fertility Rate Models, Reported as Incidence Rate Ratios.

## Data Availability

All data can be accessed on request from MDH and downloaded from the US Census Bureau at https://www.census.gov/programs-surveys/acs/.

## Abbreviations

ACS: American Community Survey
ATSDR: Agency for Toxic Substances and Diseas Registry
EPA: Environmental Protection Agency
GAC: Granular activated charcoal
GFR: General fertility rate
IRR: Incidence rate ratio
MDH: Minnesota Department of Health
MPCA: Minnesota Pollution Control Agency
OR: Odds ratio
PFAS: Per- and polyfluoroalkyl substance
PFBA: Perfluorobutyrate
PFOA: Perfluorooctanoic acid
PFOS: Perfluorooctane sulfonic acid
TTP: Time to pregnancy
