# Supplementary material for: Reducing exposure to high levels of perfluorinated compounds in drinking water improves reproductive outcomes: evidence from an intervention in Minnesota
Source: Environ Health. 2020 Apr 22;19:42. doi: 10.1186/s12940-020-00591-0 (PMC7178962; doi:10.1186/s12940-020-00591-0)
Supplement: Supplementary file 1 — Additional file 1: Figure A1. Map of Study Area and PFAS Source Locations. [file 12940_2020_591_MOESM1_ESM.docx]

**Figure A1. Map of Study Area and PFAS Source Locations**

=
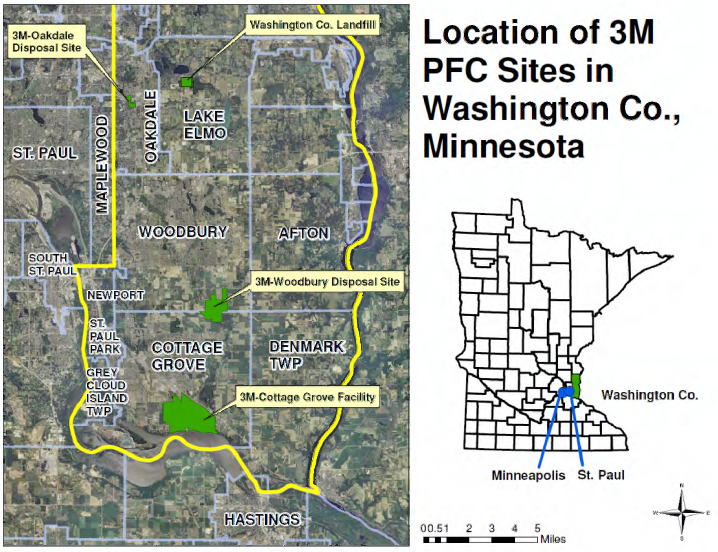


Copied from “Perfluorochemicals (PFCs) in the East Metro”, presentation prepared by James Kelly and Karla Peterson, Minnesota Department of Health, Environmental Health Division, August 21-22 2018. Accessed online at https://3msettlement.state.mn.us/sites/default/files/PFCs%20in%20the%20East%20Metro.pdf
