# Supplementary material for: Reducing exposure to high levels of perfluorinated compounds in drinking water improves reproductive outcomes: evidence from an intervention in Minnesota
Source: Environ Health. 2020 Apr 22;19:42. doi: 10.1186/s12940-020-00591-0 (PMC7178962; doi:10.1186/s12940-020-00591-0)
Supplement: Supplementary file 2 — Additional file 2: Figure A2. Current Extent of PFOS and PFOA Contamination of Groundwater in East Metro Area. [file 12940_2020_591_MOESM2_ESM.docx]

**Figure A2. Current Extent of PFOS and PFOA Contamination of Groundwater in East Metro Area**


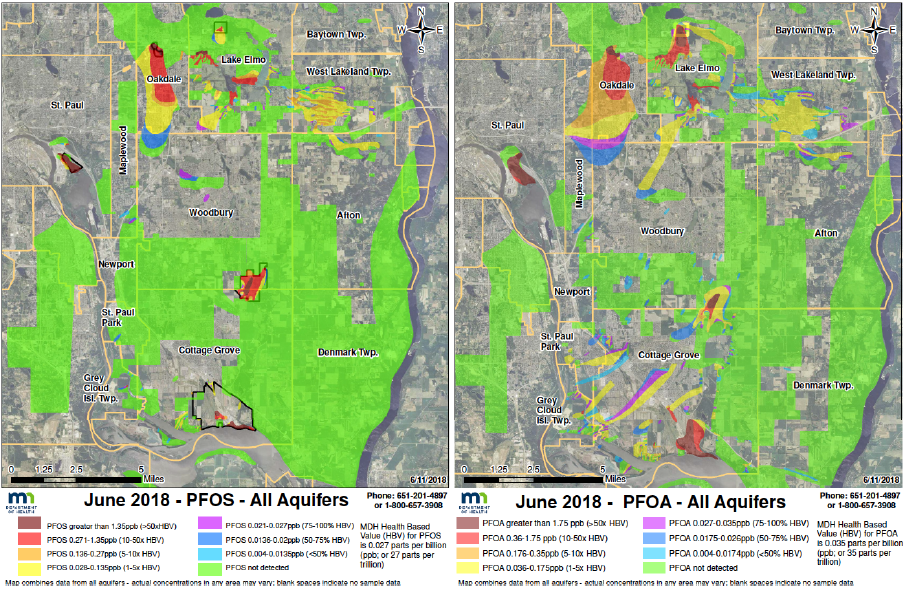


Copied from “PFOS in East Metro - 2018-2016 (PDF)”, Minnesota Department of Health, and “PFOA in the East Metro - 2018-2016 (PDF)”, Minnesota Department of Health. Accessed online at <https://www.health.state.mn.us/communities/environment/hazardous/docs/pfas/pfoseastmetro.pdf> and <https://www.health.state.mn.us/communities/environment/hazardous/docs/pfas/pfoaeastmetro.pdf>.
