# Supplementary material for: Reducing exposure to high levels of perfluorinated compounds in drinking water improves reproductive outcomes: evidence from an intervention in Minnesota
Source: Environ Health. 2020 Apr 22;19:42. doi: 10.1186/s12940-020-00591-0 (PMC7178962; doi:10.1186/s12940-020-00591-0)
Supplement: Supplementary file 3 — Additional file 3: Table A1. All Regression Coefficients for Continuous Birth Outcomes. [file 12940_2020_591_MOESM3_ESM.docx]

Table A1. All Regression Coefficients for Continuous Birth Outcome Models

All models estimated by ordinary least squares, with standard errors clustered at the zip-code level (reported in parentheses). *** p<0.01, ** p<0.05, * p<0.1. Results are qualitatively similar to those with log-transformed outcome variables.
