# Supplementary material for: Reducing exposure to high levels of perfluorinated compounds in drinking water improves reproductive outcomes: evidence from an intervention in Minnesota
Source: Environ Health. 2020 Apr 22;19:42. doi: 10.1186/s12940-020-00591-0 (PMC7178962; doi:10.1186/s12940-020-00591-0)
Supplement: Supplementary file 4 — Additional file 4: Table A2. All Regression Coefficients for Low Birth Weight (< 2500 g) and Very Low Birth Weight (< 1500 g) Models, Reported as Odds Ratios. [file 12940_2020_591_MOESM4_ESM.docx]

Table A2. All Regression Coefficients for Low Birth Weight (<2,500 g) and Very Low Birth Weight (<1,500g) Models, Reported as Odds Ratios

All models estimated by logistic regression, with standard errors clustered at the zip-code level (reported in parentheses). *** p<0.01, ** p<0.05, * p<0.1.
