# Supplementary material for: Reducing exposure to high levels of perfluorinated compounds in drinking water improves reproductive outcomes: evidence from an intervention in Minnesota
Source: Environ Health. 2020 Apr 22;19:42. doi: 10.1186/s12940-020-00591-0 (PMC7178962; doi:10.1186/s12940-020-00591-0)
Supplement: Supplementary file 5 — Additional file 5: Table A3. All Regression Coefficients for Pre-term Births (< 37 weeks) and Early Pre-term Births (< 32 weeks) Models, Reported as Odds Ratios. [file 12940_2020_591_MOESM5_ESM.docx]

Table A3. All Regression Coefficients for Pre-term Birth (< 37 weeks) and Early Pre-term Birth (< 32 weeks) Models, Reported as Odds Ratios

All models estimated by logistic regression, with standard errors clustered at the zip-code level (reported in parentheses). *** p<0.01, ** p<0.05, * p<0.1.
