# Supplementary material for: Reducing exposure to high levels of perfluorinated compounds in drinking water improves reproductive outcomes: evidence from an intervention in Minnesota
Source: Environ Health. 2020 Apr 22;19:42. doi: 10.1186/s12940-020-00591-0 (PMC7178962; doi:10.1186/s12940-020-00591-0)
Supplement: Supplementary file 6 — Additional file 6: Table A4. All Regression Coefficients for General and Age Group-Specific Fertility Rate Models, Reported as Incidence Rate Ratios. [file 12940_2020_591_MOESM6_ESM.docx]

Table A4. All Regression Coefficients for General and Age Group-Specific Fertility Rate Models, Reported as Incidence Rate Ratios

All models estimated using Poisson regression with robust standard errors (reported in parentheses). Coefficients reported as incidence rate ratios. *** p<0.01, ** p<0.05, * p<0.1.
